# The quality of free antenatal and delivery services in Northern Sierra Leone

**DOI:** 10.1186/s12961-017-0218-4

**Published:** 2017-07-12

**Authors:** Manso M. Koroma, Samuel S. Kamara, Evelyn A. Bangura, Mohamed A. Kamara, Virgil Lokossou, Namoudou Keita

**Affiliations:** 1 Department of Environmental Sciences, Makeni University College, Ernest Bai Koroma University of Science and Technology, Makeni, Sierra Leone; 2 Primary Health Care and Health Systems Strengthening Unit, West African Health Organisation, Bobo Dioulasso, Burkina Faso

## Abstract

**Background:**

The number of maternal deaths in sub-Saharan Africa continues to be overwhelmingly high. In West Africa, Sierra Leone leads the list, with the highest maternal mortality ratio. In 2010, financial barriers were removed as an incentive for more women to use available antenatal, delivery and postnatal services. Few published studies have examined the quality of free antenatal services and access to emergency obstetric care in Sierra Leone.

**Methods:**

A cross-sectional survey was conducted in 2014 in all 97 peripheral health facilities and three hospitals in Bombali District, Northern Region. One hundred antenatal care providers were interviewed, 276 observations were made and 486 pregnant women were interviewed. We assessed the adequacy of antenatal and delivery services provided using national standards. The distance was calculated between each facility providing delivery services and the nearest comprehensive emergency obstetric care (CEOC) facility, and the proportion of facilities in a chiefdom within 15 km of each CEOC facility was also calculated. A thematic map was developed to show inequities.

**Results:**

The quality of services was poor. Based on national standards, only 27% of women were examined, 2% were screened on their first antenatal visit and 47% received interventions as recommended. Although 94% of facilities provided delivery services, a minority had delivery rooms (40%), delivery kits (42%) or portable water (46%). Skilled attendants supervised 35% of deliveries, and in only 35% of these were processes adequately documented. None of the five basic emergency obstetric care facilities were fully compliant with national standards, and the central and northernmost parts of the district had the least access to comprehensive emergency obstetric care.

**Conclusion:**

The health sector needs to monitor the quality of antenatal interventions in addition to measuring coverage. The quality of delivery services is compromised by poor infrastructure, inadequate skilled staff, stock-outs of consumables, non-functional basic emergency obstetric care facilities, and geographic inequities in access to CEOC facilities. These findings suggest that the health sector needs to urgently investigate continuing inequities adversely influencing the uptake of these services, and explore more sustainable funding mechanisms. Without this, the country is unlikely to achieve its goal of reducing maternal deaths.

## Background

Sub-Saharan Africa stands out as the region in the world with the slowest rate of decline of maternal deaths between 1990 and 2015, averaging a mere 2.3% annual decline. The region alone accounts for 66% of global maternal deaths [1]. All of the 18 countries with the highest maternal mortality ratio (MMR) in 2015 are in Africa.

Sierra Leone tops this list with a MMR estimated at 1360 per 100,000 (UI 999–1908) [1]. This rate is similar to the natural MMR estimated at approximately 1000–1500 per 100,000 found in situations where no interventions are used to avert maternal deaths [2]. Pregnant women in Sierra Leone have a higher chance of dying, an approximate lifetime risk of maternal mortality of 1 in 17, compared to women in high-income countries who have a risk of 1 in 3300 [1].

**Fig. 1:**
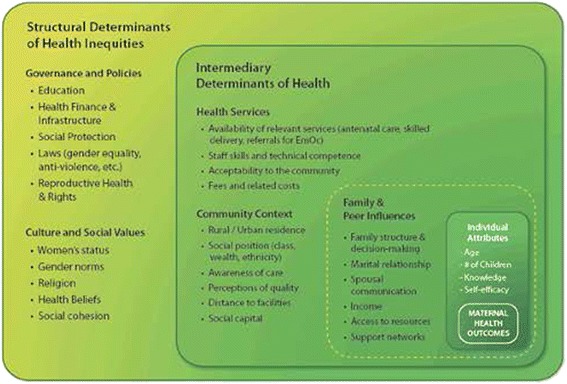
The social determinants of maternal health. Adapted from: WHO (2011) Closing the gap: policy into practise on social determinants of health [3]

In sub-Saharan African countries, this lack of progress in reducing maternal deaths reflects persisting inequities between social groups that are perpetuated by formal social structures and institutions, socioeconomic factors, as well as cultural and social practices (Fig. 1). These structural inequities influence the extent to which women in different social classes can readily access and use maternal health services at an adequate level of quality [3, 4].

Women who are socially or economically disadvantaged and geographically isolated have an increased risk of dying during pregnancy, even though most of these deaths are largely preventable if they receive interventions in time [2, 5]. For instance, poorer women in sub-Saharan African countries lack timely access to both skilled attendants at delivery and institutional delivery care services, unlike in rich countries, where the skills to use life saving devices and to carry out emergency procedures (caesarean section, blood transfusion and effective antibiotics) are widely available [6]. When inequities in accessing delivery services are reduced, there are fewer maternal deaths. After Rwanda expanded access to institutional deliveries between 1990 and 2015, the proportion of women delivered by skilled birth attendants increased over three-fold, from 25.8% to 90.7%, and the MMR declined by 78% in the same period [1, 7, 8].

Aside from health system determinants, household income contributes to maternal mortality [9]. In low-income countries, poor women are unwilling to use the formal health sector if they must pay for maternal health services [10]. This is an important observation as countries that successfully lowered maternal deaths also improved financial access to professional care [5, 6]. There is evidence of a rise in facility births and reduced deaths among newborns following the removal of user fees [10, 11]. In the face of such strong evidence governments in developing countries were urged to put in place maternal fee exemptions as a strategy to reduce maternal deaths. Thus far, 15 sub-Saharan African countries have abolished fees [10].

**Fig. 2:**
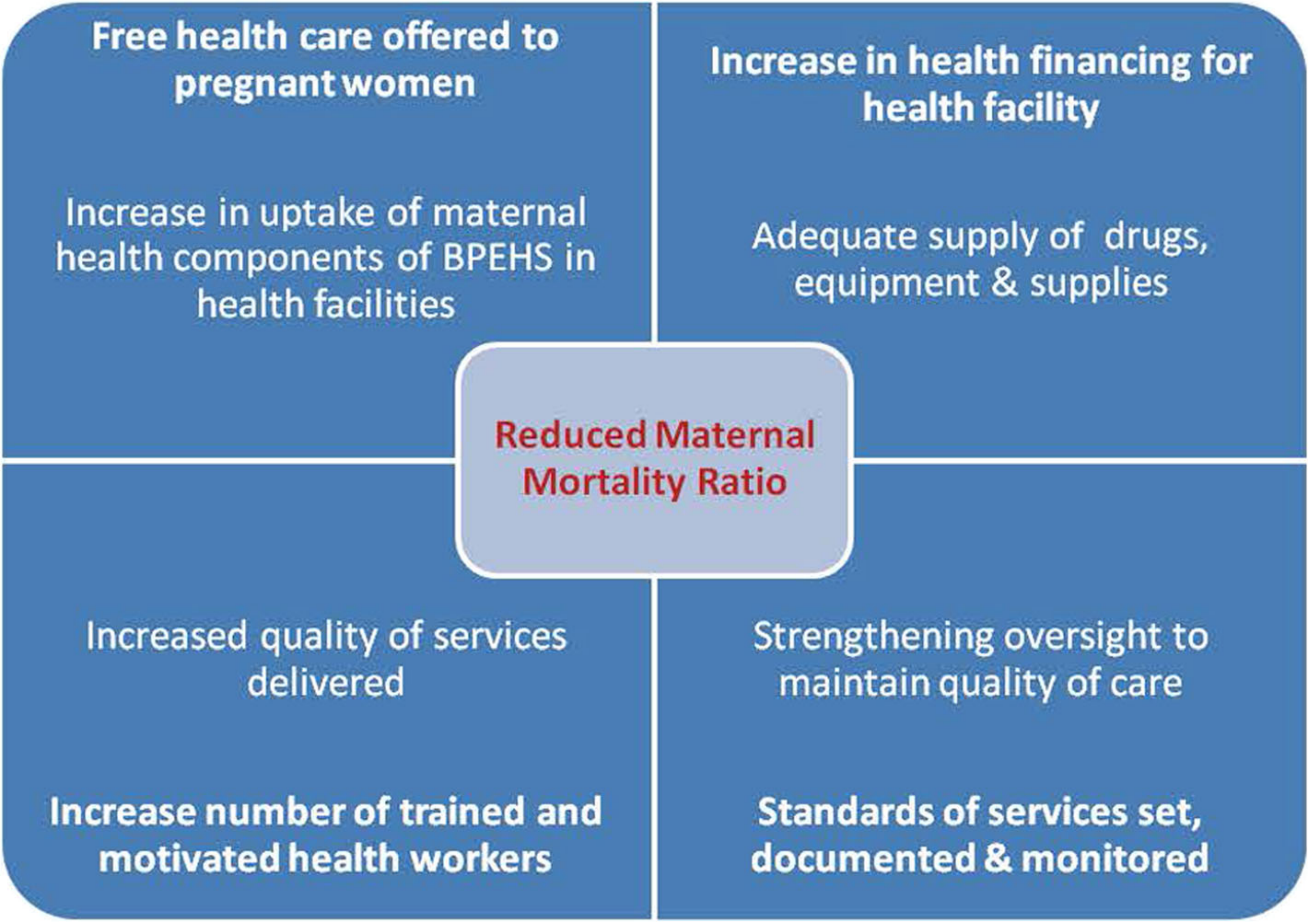
Conceptual model of the Free Health Care Initiative in health facilities

In 2010, the government of Sierra Leone launched the Free Health Care Initiative (FHCI) [12] that exempted pregnant women, breastfeeding mothers and children under 5 years of age from paying fees for services. The conceptual framework in Fig. 2 below explains how the FHCI is expected to work to reduce maternal deaths in health facilities.

It is assumed that, if more pregnant women use the free maternal services and if the quality of these services are improved and sustained by a motivated and competent workforce, then maternal health outcomes would improve and the MMR would fall. The FHCI was examined from the outset and several studies were conducted to assess its effectiveness. However, these studies were mainly qualitative in nature, involved relatively few health facilities or focussed on selected aspects of the FHCI, and did not produce adequate data at the decentralised level to determine how well the initiative was working [13–16].

This larger study was designed to generate more data to inform decision-making at the sub-national level, which is important given the on-going decentralisation reforms in the country. The objectives of the study were to investigate the quality of antenatal and delivery services provided within health facilities implementing the FHCI in Bombali district, Northern Sierra Leone, and to identify solutions to overcome identified barriers that are preventing the FHCI from being delivered effectively within districts.

## Methods

### Study design and population

The cross-sectional health facility survey was conducted from March to April 2014 in Bombali District, one of five districts in the Northern Region of Sierra Leone. The survey included all public and missionary health facilities in the district. In total, 100 health facilities were surveyed, consisting of three district hospitals, 94 public peripheral health units and three not-for-profit health facilities. The survey team interviewed the officers-in-charge on the day of the survey and observed two or three antenatal providers at work within each of the 97 peripheral health units. Face-to-face interviews were conducted with 486 pregnant women who accessed antenatal services in the surveyed health facilities on the day of the study. They were interviewed immediately after they received antenatal services.

### Data collection

We adapted and field tested the WHO safe motherhood questionnaires and the antenatal care observation checklist developed by the USAID Maternal and Child Health Integrated programme [17, 18]. We collected data on the numbers of beds assigned to pregnant women in facilities; the infrastructure, equipment, drugs and supplies of the clinic; antenatal and delivery services; complications that occurred; laboratory services; emergency obstetric services and referrals; and family planning and educational materials in the clinic. We observed and assessed current practice in antenatal clinics using the antenatal checklist.

In the antenatal client exit interview, women were asked about their age, how they got to the clinic, the cost of using the services, their maternity and delivery history, the services and counselling they received in the clinic, and their knowledge of pregnancy-related danger signs. Several records, including antenatal cards, delivery register, normal delivery and complicated delivery records, were reviewed retrospectively. Data were collected on maternal and newborn outcomes, including mode of delivery, live births, Apgar scores, birth weight, fresh stillbirths, macerated stillbirths, immediate neonatal deaths, maternal outcome and referrals.

### Data analysis

Quantitative data were analysed using SPSS. The summary measures were proportions calculated for quantitative variables. Confidence intervals were calculated for proportions. National standards [19] for antenatal interventions in peripheral health units during antenatal clinics were adapted and used to assess the adequacy of antenatal services offered to women in three domains. A woman who was examined for the recommended six physical signs on her first antenatal visit was rated as having received an adequate examination. If fewer signs were examined for, she was rated as having been inadequately examined. Women who were observed to receive the four recommended basic tests on their first antenatal visit were adequately screened and fewer tests were rated as inadequately screened. If three interventions were offered to women in their third trimester, women were considered to have received adequate interventions. Fewer interventions were considered an inadequate level of care.

The distance was calculated between each facility providing delivery services and the nearest comprehensive emergency obstetric care (CEOC) facility. Then, the proportion of facilities in a chiefdom within 15 km of each CEOC facility was calculated. A thematic map was developed to show inequities in CEOC facility service provision.

The study was approved by the National Ethics and Scientific Review Committee of the Ministry of Health and Sanitation. Pregnant women who participated gave individual informed consent.

### Study limitations

The outbreak of Ebola in May 2014 made it difficult to collect qualitative data. Focus group discussions were not convened as planned because of the Health Emergency Regulation limiting the movement of people. The study did not collect data on the structural and intermediary determinants of heath inequities that affect maternal outcomes.

## Results

**Fig. 3:**
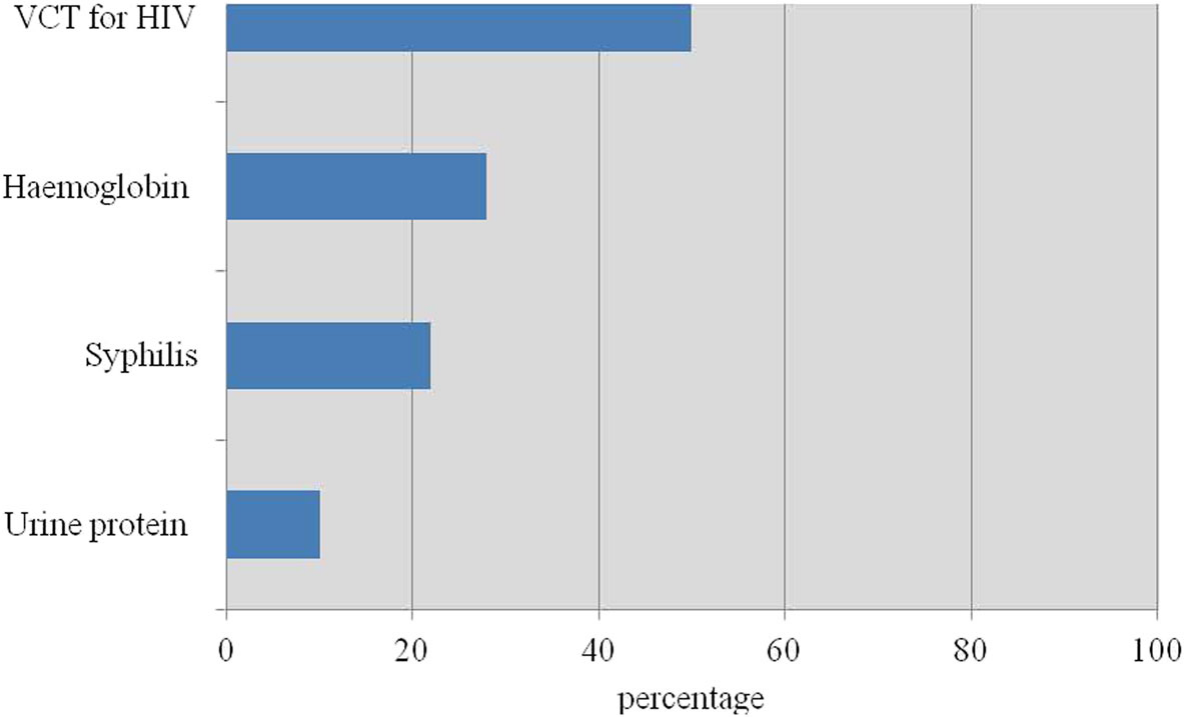
Percentage of women observed receiving adequate or inadequate level of antenatal services in health facilities

Physical access to antenatal and delivery services was widespread. Out of the 97 public facilities surveyed, 95% provided daily antenatal services, 94% provided delivery services and 86% provided postnatal services. Of these facilities, 21% were Community Health Centres, 38% were Community Health Posts and 40% were Maternal and Child Health Posts. Of the 486 pregnant women interviewed, 93% received free care during their last pregnancy. We found that the quality of the services they received varied (Fig. 3). Based on national standards, only 27% of women were examined, 2% were screened and 47% received interventions as recommended.

Less than a third of women were screened for haemoglobin estimation, albumin in urine and syphilis (10–28%), compared to an HIV test (50%) (Table 1). Most antenatal health providers (over 80%) were observed to weigh, measure blood pressure, palpate the abdomen and listen to the foetal heart. However, fewer health providers checked for oedema (45%) and anaemia (52%) (Table 1). Iron prophylaxis was the most common intervention given to women, whereas fewer women (65%) recalled being adequately counselled on all four topics related to birth preparedness (Table 2). There were stock-outs of some essential antenatal drugs, consumables and equipment needed for antenatal assessment. Laboratory consumables were more likely to be unavailable (86–97%) compared to equipment (13.3–47.4%) or medication (12.4%) (Table 3). Even though only 62% of the 244 antenatal women recalled being counselled on all four topics, recall rates were high for individual topics (Table 4).

**Table 1:** Percentage of women on their first antenatal visit who were screened and examined for different physical signs

	Pregnant women seen in third trimester % (*n* = 58)	95% CI
Screening test		
Urine protein	10	2.5–18.2
Syphilis	22	11.7–33.2
Haemoglobin	28	16.1–39.1
Voluntary counselling and testing for HIV	50	37.1–62.9
Physical signs		
Check for oedema	45	32.0–57.6
Check for anaemia	52	38.9–64.6
Listen to foetal heart	76	64.9–86.9
Weight	79	68.9–89.7
Abdominal palpation	79	68.9–89.7
Measure blood pressure	83	73.0–92.5

**Table 2:** Percentage of women in the third trimester of pregnancy who received antenatal interventions

Intervention	Pregnant women seen in third trimester % (*n* = 244)	95% CI
Information, education and communication of birth preparedness	65	58.8–70.8
Fansidar/sulfadoxine-pyrimethamine given for intermittent preventive treatment	89	85.5–93.2
Iron/folic acid	98	96.1–99.7

**Table 3:** Percentage of health facilities with stock-out of antenatal equipment, drugs and supplies

Item	Facilities (n)	%	95% CI
Syphilis test kit	95	96.8	92.5–100
Urine test kit	93	86	78.5–92.5
Blood pressure machine	97	47.4	37.1–57.7
Fetal stethoscope	97	15.5	8.3–22.7
Partogram	90	13.3	6.7–21.1
Iron tablets	89	12.4	5.6–19.1

**Table 4:** Percentage of pregnant women in the third trimester who received advice on birth preparedness

Topic discussed	Pregnant women seen in third trimester (*n* = 244)	
	%	95% CI
Place of birth	95	92–97
What to do if there is a problem during the pregnancy	87	83–91
Benefit of birth in a health facility	82	77–86
How to get to the health facility in an emergency	82	76–86

Delivery facilities lacked essential infrastructure, equipment, drugs and supplies. Of the 97 facilities surveyed, 60% had no delivery room, 54% had no portable water, 58% lacked the minimum equipment needed for deliveries and 79% had no table and stool for gynaecological examinations. Only 35% of delivery records adequately documented processes during labour, and just over a third of women were attended by a skilled attendant (midwife, doctor, nurse) (Table 5).

**Table 5:** Percentage of births attended by a skilled health provider

Cadre	Women who delivered in a health facility at last pregnancy (*n* = 340)	
	%	95% CI
Nurse or midwife	35.6	30.6–40.6
Doctor or clinic officer	1.8	0.6–3.2
Maternal and child health aide	56.5	51.2–61.8
Traditional birth attendant	7.4	4.7–10.3
Family member/other	5.9	3.5–8.5

Among the 97 facilities surveyed, five Community Health Centres were designated as basic emergency obstetric care facilities. Based on national standards, these facilities are supposed to stock life-saving drugs and have life-saving skills, including neonatal resuscitation. None of the five basic emergency obstetric care facilities were fully compliant with national standards (Table 6) nor had all the necessary skills to manage emergencies commonly seen in the district (infection, haemorrhage, retained placenta, pre-eclampsia and eclampsia, severe anaemia, breech presentation).

**Table 6:** Percentage of basic emergency obstetric care facilities that met national standards

Assessed standard	Percentage of facilities that met standards (*n* = 5)
Electricity supply	100
Water supply	80
Drugs and consumables	40
Equipment	20
Functional referral system	0
Functioning laboratory	0

**Fig. 4:**
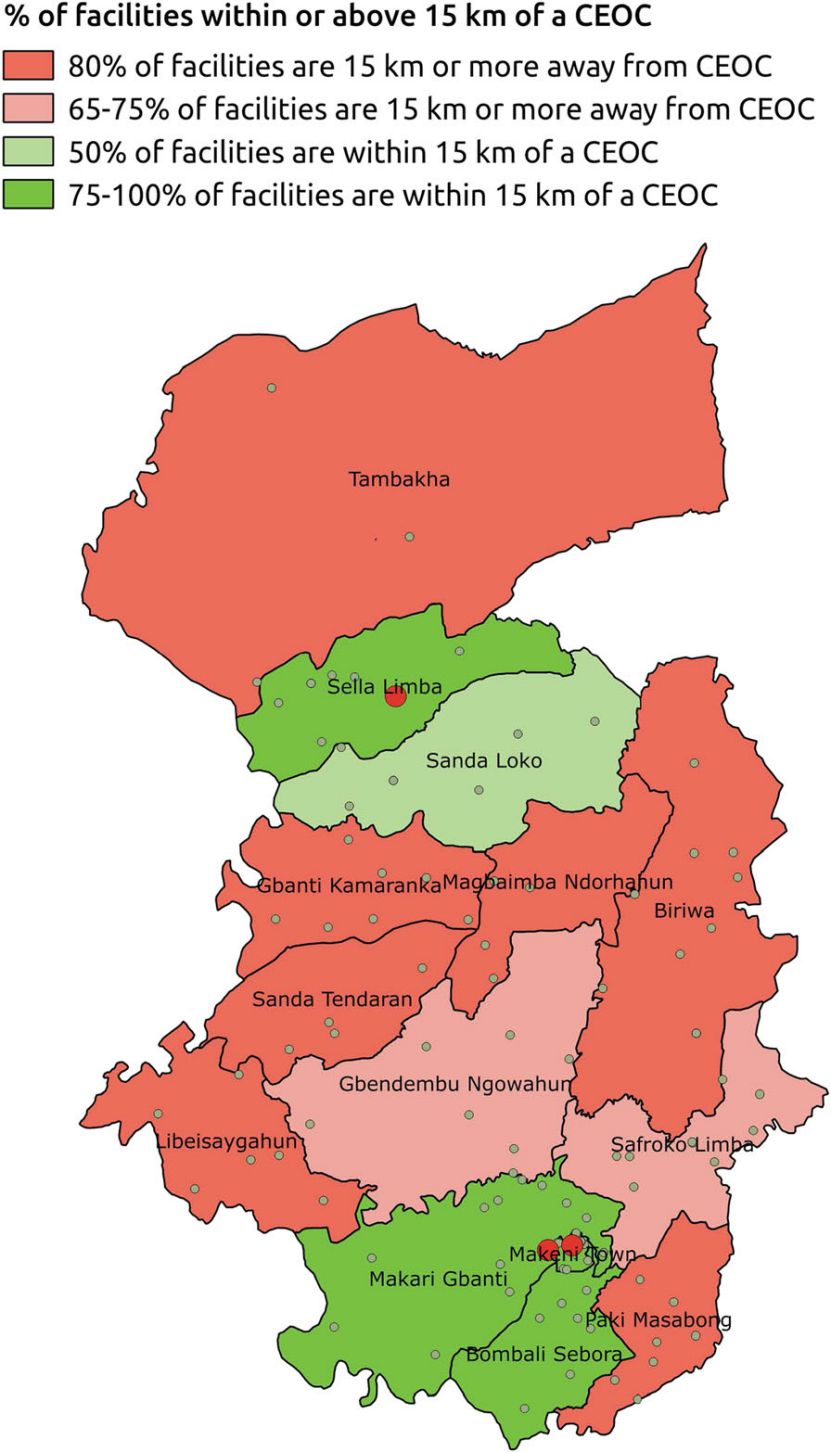
Percentage of facilities within 15 km of a comprehensive emergency obstetric care facility

Although the three district hospitals met national standards for CEOC facilities, there were inequities in accessing these services. Spatial analysis revealed that the central and northernmost parts of the district had the least access to these services (Fig. 4)

## Discussion

Free maternity services are promoted as a strategy to improve maternal outcomes in sub-Saharan African countries [20]. However, despite the willingness of women in this study to use the free maternal health services in northern Sierra Leone, there were several constraints resulting in sub-standard maternity services, especially delivery care.

There is evidence that, following the launch of free antenatal services, the uptake of antenatal care may have initially improved among subgroups. Antenatal coverage for at least four visits increased from 56% in 2008 to 75% in 2010 after the launch of free services in Sierra Leone, and marginally to 76% in 2013 [21–23]. Although the upward trend in antenatal visits was evident among all sub-groups of women, the greatest increase in uptake between 2008 and 2013 was seen among previously disadvantaged subgroups, namely women with no education, those that reside in rural areas and those in the lowest wealth quintile, and among those in the regions with the lowest percentage of women with four or more antenatal care visits in 2008 [24].

Despite this change, as described in this study and in other African countries, women receive suboptimal services [25–27]. This is of concern because it deprives women from receiving interventions that can improve their health, increase their awareness of potential complications and birth preparedness, and screen for conditions that could affect the newborn [6, 28]. Women who become discouraged because of the low quality of care they receive may also opt out of using the formal health sector and decrease their likelihood of receiving professional care during the perinatal period when they are most vulnerable and when most women die [29]. The findings show the need for the health sector to monitor both antenatal coverage rates and the quality of care offered to women. However, it is unclear what indicators should be used to assess quality of antenatal services in the absence of an agreed framework for low resource countries [26]. More work is needed to define a set of indicators for resource-constrained settings, as this lack of consensus makes it difficult to monitor trends and to directly compare the outcome of studies based on different measures of quality of antenatal care.

This is less of a problem with emergency obstetric care, where international norms exist. We found that the emergency obstetric facility coverage rate of 8 per 500,000 people exceeded the international norm of at least five emergency obstetric care facilities, including at least one comprehensive facility per 500,000 population. Yet, the overall quality of emergency obstetric care was low. Previous surveys in 2011 and 2012 of health facilities in Sierra Leone found that just under a third of facilities were qualified to provide basic emergency obstetric care [30, 31]. If women cannot have access to emergency interventions when complications occur, it is unlikely that preventable maternal deaths would be averted [32]. This implies that mere physical availability is insufficient without regard to how easily facilities can be reached and whether they are fully functioning. Spatial tools can be useful in identifying poorly served geographical areas [33, 34].

For both emergency care and antenatal services, it could be argued that the quality of services that depend on external inputs, such as equipment, laboratory consumables and drugs, would deteriorate if there are regular stock-outs of these items as previously described [30, 31]. However, this is less likely to affect externally funded primary healthcare interventions that rely on more consistent inputs. This may account for the observed discrepancy seen in this study between the larger number of women screened for HIV as part of a national donor funded programme compared to the number of women screened for other conditions funded by the health sector.

What was surprising was how poorly health providers performed, even in instances where inputs were unnecessary. In this study, antenatal health providers did not consistently fully examine women for important physical signs at different stages of their pregnancy, nor did they counsel women on the full range of relevant topics, including voluntary counselling and testing to prevent mother-to-child transmission of HIV/AIDS, as well as birth preparedness. One limitation of this study was that data was not collected to explore why this was so. Low morale due to increased workload may be one factor as the number of health providers providing free maternal services has not increased concurrently to provide full coverage to meet the rising demand for delivery services and emergency care [35, 36]. This has negatively affected the quality of maternity services [35].

Some suggest that, in the short-term, the workforce should be expanded and adequately compensated to cope with the growing demand on the health sector. However, there is also growing awareness that the health sector needs to make health providers more accountable for actions inside the health system that perpetuate maternal mortality [8]. There are calls to set up systems that foster a culture of quality of care sustained by a system of control and professional accountability through mechanisms such as increasing public demand for good quality care, maternal audits, confidential enquiries and mentorship programmes [6, 8, 36]. With these policies in place and enforced, it is feasible, as seen in Rwanda, for maternal deaths to fall substantially in African countries [8]. However, whilst better health governance can and does contribute to improving maternal health outcomes, existing entrenched socioeconomic inequities beyond the health sector may still undermine these efforts to improve maternal health [37]. Another limitation of this study was that it did not explore and analyse data on social determinants of maternal health and how they affect the uptake of interventions such as free maternal services. There is a need to better understand why and how structural inequities in countries with high maternal mortality rates continue to hinder efforts to reduce them.

If the quality of free maternal services is low, uptake will fall. This is already apparent in some sub-Saharan African countries [38]. At the same time, the identified gaps in quality can only be reduced if governments in low-income countries generate and sustain adequate long-term funding to cover investments to maintain infrastructure and equipment, provide a regular supply of drugs and consumables, and meet the salaries of an increasing workforce [39]. Free maternal services are costly. The government of Sierra Leone and partners committed US$91 million when the FHCI was launched in 2010, with a funding gap of US$20 million [12]. Government spending on the health sector as a proportion of total national expenditure declined from 12% in 2010 to 9.8% in 2014, below the agreed Abuja target of 15% [40]. If poor countries like Sierra Leone are struggling to finance their health sector, the challenge is to determine how and where additional investments will be found to urgently improve the quality of free maternal services. Further work needs to be done to investigate structural inequities that perpetuate maternal mortality and to explore strategies to sustainably fund free maternity services at an adequate level of quality. Without this, the gains made so far could be reversed and maternal deaths may once again increase to unacceptably high levels.

## Conclusion

Although antenatal coverage is high in the surveyed regions, we found evidence of substandard antenatal services. The findings lend weight to the need to routinely monitor not just coverage, but also the quality of services offered to women. Although the lack of external inputs was partly responsible for the poor quality of both antenatal and delivery services, this could not account for the observed poor performance of health workers. More in-depth qualitative assessments are needed to better understand how to improve professional accountability in providing health services, including maternal health services. The health sector needs to urgently invest more resources to explore how to sustainably finance free maternal services at an adequate level of quality and to investigate continuing inequities adversely influencing the uptake of these services towards reaching its goal of reducing maternal deaths.
